# A PSO-Based Uneven Dynamic Clustering Multi-Hop Routing Protocol for Wireless Sensor Networks

**DOI:** 10.3390/s19081835

**Published:** 2019-04-17

**Authors:** Danwei Ruan, Jianhua Huang

**Affiliations:** School of Information Science and Engineering, East China University of Science and Technology, Shanghai 200237, China; rdw1226@163.com

**Keywords:** particle swarm optimization, clustering, routing protocol, wireless sensor networks

## Abstract

Since wireless sensor networks (WSNs) are powered by energy-constrained batteries, many energy-efficient routing protocols have been proposed to extend the network lifetime. However, most of the protocols do not well balance the energy consumption of the WSNs. The hotspot problem caused by unbalanced energy consumption in the WSNs reduces the network lifetime. To solve the problem, this paper proposes a PSO (Particle Swarm Optimization)-based uneven dynamic clustering multi-hop routing protocol (PUDCRP). In the PUDCRP protocol, the distribution of the clusters will change dynamically when some nodes fail. The PSO algorithm is used to determine the area where the candidate CH (cluster head) nodes are located. The adaptive clustering method based on node distribution makes the cluster distribution more reasonable, which balances the energy consumption of the network more effectively. In order to improve the energy efficiency of multi-hop transmission between the BS (Base Station) and CH nodes, we also propose a connecting line aided route construction method to determine the most appropriate next hop. Compared with UCCGRA, multi-hop EEBCDA, EEMRP, CAMP, PSO-ECHS and PSO-SD, PUDCRP prolongs the network lifetime by between 7.36% and 74.21%. The protocol significantly balances the energy consumption of the network and has better scalability for various sizes of network.

## 1. Introduction

Wireless sensor networks (WSNs) have attracted widespread attention in recent years. Due to the low cost, small size and self-organization of sensors [1], WSNs have been adopted in diverse application fields, such as military, crime prevention, environmental monitoring, health care services, vehicular movements, etc. [2,3,4] As sensor nodes are supplied by non-rechargeable batteries [5], designing an energy-efficient routing protocol to prolong the network lifetime is a vital issue in WSNs.

A number of routing protocols have been proposed to reduce the energy consumption of WSNs. Among them, the clustering scheme has better flexibility and scalability, and is considered to be one of the most effective solutions in this regard. Therefore, most of the current researches on routing protocols are based on the clustering scheme, such as LEACH (Low-Energy Adaptive Clustering Hierarchy) [6], PEGASIS(Power-Efficient GAthering in Sensor Information Systems) [7], HEED (Hybrid Energy-Efficient Distributed clustering) [8], EEMC (Energy-Efficient Multi-level Clustering) [9] and so on. Clustering can improve energy efficiency, but it can cause hotspot problems [10,11].

Many energy-efficient clustering routing algorithms have been proposed to solve the hotspot problem. Reference [12] proposed a gravitational search algorithm (GSA) based clustering and routing algorithm. GSA uses formulae to elect CH nodes and assign nodes to address the hotspot problem. The UCCGRA (Unequal Clustering and Connected Graph Routing Algorithm) algorithm [13] considers the clustered network with unequal size based on sensor energy used for the transmission. The work highlights the concept of balancing the node energy for inter and intra cluster communication. In UCCGRA, the vote based selection of CH nodes creates more control message overheads during re-clustering, which cause unnecessary energy depletion. Grid-based clustering protocols, such as [14,15,16], form clusters by dividing the network area into grids. The size of the grids away from the BS is larger than the size of the grids near the BS, which can alleviate the hotspot problem to some extent. However, for a network environment with uneven node distribution, grid-based clustering is unreasonable. Because there may be a large difference in the number of nodes in the same size grids.

The aforementioned protocols improve the energy efficiency of WSNs. However, not all the protocols carefully consider the distribution of nodes. Moreover, most of these algorithms focus only on the selection of CH nodes. They do not carefully consider the distribution and scale of clusters. The protocols suffer from an energy imbalance across the network. The hotspot problem still exists to a certain extent and some protocols cannot be applied to large-area network environments. This paper proposes a PSO-based uneven dynamic clustering multi-hop routing protocol (PUDCRP), which alleviates the hotspot problem and achieves better energy balance. We use an improved PSO algorithm to determine the circular area where candidate CH nodes are located. We introduce a multi-objective fitness function to select CH nodes. We also propose a connecting line aided route construction method to achieve an energy efficient routing. Simulation results proved that PUDCRP has a better performance in network lifetime and energy consumption of the network. The major contributions of this paper can be summarized as follows.

- We propose a PSO-based uneven dynamic clustering method which divides the network area into circles with unequal sizes based on the distribution of nodes. The circles far away from the BS are larger than the ones near the BS, which can alleviate the hotspot problem.
- We introduce a multi-objective function to select CH nodes from the circles. The function considers nodes’ residual energy, the number of neighbor nodes, and distances from the nodes to the BS. Therefore, the intra-cluster energy consumption is minimized.
- Two fitness functions are proposed to determine the optimal positions of the circular areas in our PSO method. The *G_best_* fitness function is to achieve the maximum coverage across the network. The fitness function used to determine *P_besti_* is the absolute value of the difference between the actual number of nodes covered by the circular area and the number of ideal coverage nodes, which makes each circular area contain the number of nodes that match its size.
- A connecting line aided route construction method is proposed to determine a multi-hop route. The method considers the distance from the candidate CH node to the connecting line between the source CH node and the BS, the transmission distance from the source CH node to the candidate CH node, and the residual energy of candidate CH node. Hence, the energy consumption of the transmission is reduced.

The rest of the paper is organized as follows. The related work is described in Section 2. The network and energy models are proposed in Section 3. The overview of PSO is introduced in Section 4. Section 5 introduces the detailed PUDCRP protocol. Experiment results are discussed in Section 6 by comparing with other protocols. The conclusion is presented in Section 7.

## 2. Related Work

### 2.1. Classical Routing Protocols

Low energy adaptive clustering hierarchy (LEACH) [6] is a well-known clustering protocol. An important contribution of LEACH is the introduction of the concept of clustering hierarchy for load balancing among sensor nodes. The operation of LEACH is divided into a number of rounds. Nodes take turns as CH nodes to balance the energy consumption of the nodes. The main drawback of the LEACH algorithm is that random selection of CH nodes may result in uneven distribution of them and cause the imbalance of energy consumption. Moreover, the single-hop transmission of LEACH causes the CH nodes to quickly consume energy and die sooner. There have been many improvements [17] to the shortcomings of LEACH. CogLEACH (Cognitive LEACH) [18] improved the LEACH deficiency to make the CH distribution more reasonable. Aiming at the problem of uneven energy consumption of CogLEACH, Latiwesh et al. [19] introduced a centralized cognitive LEACH (CogLEACH-C). In the protocol, besides idle channels, nodes’ residual energy is also used as a parameter for the CH selection to balance the energy load of nodes. However, it is not suitable for large-scale networks because it uses the single-hop transmission during routing. Arumugam et al. [20] proposed a new idea called EE-LEACH (Energy-Efficient LEACH protocol). It provides an optimal cluster formation and efficient data aggregation, which saves a significant amount of energy. However, combination of several technologies increases the complexity of the algorithm. 

Grid-based protocols, such as EEBCDA (Energy Efficient and Balanced Cluster-based Data Aggregation algorithm) [14] and Multi-hop EEBCDA [15], can efficiently address the hotspot problem. Multi-hop EEBCDA is an improvement based on EEBCDA. It divides the network area into multiple rectangles where each rectangle has an unequal number of grids and the nodes in each grid form a cluster. This approach significantly reduces the amount of energy utilization and increases the network lifetime. Since the number of grids per rectangle is inconsistent, there is unnecessary forwarding between the layers. There may be a case where the upper layer nodes died and the lower layer nodes cannot transmit data, which means a lot of energy waste. EEMRP (Energy-Efficient Multi-hop Routing Protocol) [16] proposed a grid clustering algorithm for creating clusters and introduced communication management (CM) nodes to relay multi-hop data transmission. The method effectively balances the energy consumption of the network because the CM nodes share the workload of the CH nodes. However, the division of each grid needs to be determined in advance, which limits the scalability of the algorithm. Sajwan et al. proposed the CAMP (Cluster Aided Multi-Path) routing protocol [21]. The protocol divides the network area into equal virtual areas. Each virtual area is a cluster. In each round, a node can be either a cluster member or a separate node. The nodes perform multi-hop transmission to achieve full coverage of the network. However, CAMP divides the network area into equal areas without taking the hotspot problem into account during data transmission. Myoupo et al. [22] proposed a fault-tolerant and energy-efficient routing protocol for a virtual three-dimensional wireless sensor network. The protocol can run on any type of 3D WSNs. However, the coordinate system divides the sensor network area into equiangular wedges (or sections), which may cause hotspot problem in the network.

### 2.2. Swarm Intelligence Based Routing Protocols

Swarm intelligence has an essential idea of self-association and self-organization that can offer better solution for optimizing routing protocols of WSNs. Ari et al. [23] developed a cluster-based power efficient routing protocol named as ABC-SD. This protocol utilizes search features of artificial bee colony (ABC) which is used to design the low power consumption cluster. However, the algorithm uses a distributed method for cluster head elections, and the threshold energy used to elect CHs is fixed. If all the energy in the cluster is less than the threshold energy, CHs may not be elected. Yalçın et al. [24] proposed two algorithms, namely CH selection, based on bacterial interaction, and a cognitive routing algorithm for energy and transmission boundary. From the simulation results that were obtained from the Matlab 2016b software (MathWorks, Natick, America). It can be said that the study is more adaptive and applicable in real WSN scenarios. Karaboga et al. [25] proposed a clustering routing protocol based on an artificial bee colony algorithm in order to increase network lifetime. The algorithm employs a QoS (Quality of Service) mechanism to minimize the delays between signals received from the clusters. However, this protocol does not consider the coverage of the CHs, which leads to unbalanced energy consumption. Rao et al. [26] proposed a Particle Swarm Optimization based Energy efficient Cluster Head Selection algorithm (PSO-ECHS) which considers various parameters such as intra-cluster distance, sink distance and residual energy of sensor nodes to select CH nodes. Due to several factors being considered at the same time, some nodes far from CH nodes die prematurely when the CH node selected by the nodes has more residual energy and is far away from these nodes. Kuila et al. [27] used a PSO-based clustering algorithm to enhance the lifetime of WSNs. In this method, clustering takes place based on the average cluster distance and the lifetime of the gateway. The fitness value of each particle in the swarm is computed by using fitness function and this fitness value is used to judge the quality of the network. A particle with better fitness function value gives better network structure. Xiang et al. [28] proposed a PSO-based energy efficient routing algorithm. The fitness function of the algorithm considers the residual energy of nodes and transmission distance to balance the network energy consumption to some extent. Wang et al. [29] proposed a special clustering method called Energy Centers searching using Particle Swarm Optimization (EC-PSO) for heterogeneous WSNs. It adopted EC-PSO to elect nodes close to the energy center as CHs. However, EC-PSO is only applicable to network environments with even nodes since it uses geometric method to achieve evenly distributed CHs during the first period. Kaswan et al. [30] proposed a multi-objective and PSO based energy efficient path design for mobile sink in wireless sensor networks. The algorithm is presented with an efficient particle encoding scheme and derivation of a proficient multi-objective fitness function. Objectives include minimizing the longest path length and minimizing the number of multi-hops. However, because energy is not considered in objectives, some nodes may die prematurely. Latiff et al. [31] proposed an energy-aware clustering for wireless sensor networks using particle swarm optimization, which defines a new cost function to minimize the intra-cluster distance and optimize the energy consumption of the network. However, CHs directly transmitted data to the BS during routing. It may cause unbalanced energy consumption. Singh et al. [32] proposed particle swarm optimization (PSO) approach for generating energy-aware clusters by optimal selection of cluster heads. The PSO eventually reduces the cost of locating optimal position for the head nodes in a cluster. The PSO-based approach was implemented within clusters rather than the BS, which makes it a Particle Swarm Optimization Semi-Distributed method (PSO-SD). However, the distance between nodes and the BS are not taken into account in the protocol, which may result in excessive energy consumption of the CHs to transmit data to the BS.

## 3. Network and Energy Model

The aim of the proposed protocol in this paper is to provide the more appropriate distribution of cluster heads and the better scalability for different scale network environments, and effectively improve the energy efficiency for WSNs. We mainly consider the clustering method, the algorithm for selecting CH nodes, and the routing algorithm to balance the energy consumption and prolong the network lifetime.

### 3.1. Network Model

This paper considers randomly deploying *n* sensor nodes in a square area of size *M* × *M*, The square area is represented by symbol *A*. The assumptions of the network environment are as follows:

- All sensor nodes are homogeneous and have the same initial energy.
- Each sensor nodes are aware of its own location by using GPS (Global Positioning System) or some other localization mechanisms.
- After all the sensor nodes are deployed, they are fixed.
- All the sensor nodes are aware of their residual energy and have same transmission range.
- Sensor nodes can adjust their own energy consumption based on the distance to the receiver.
- Each node has a unique ID.
- The BS is static and positioned at the boundary of the square area.

### 3.2. Energy Model

The energy model of this paper is the same as literature [33] and [34]. The energy consumption of nodes mainly occurs at the transmitter, the power amplifier, and the receiver to run the radio electronics. The model adopts the free space and the multi-path fading channel, depending on the distance between the transmitter and receiver. The energy consumption of a node is proportional to *d*^2^ when the propagation distance *d* is less than the threshold distance *d*_0_, otherwise it is proportional to *d*^4^. The total energy expended to deliver an *l*-bit packet from the transmitter to its receiver over a link of distance *d* is calculated by Equation (1):

(1) $$E_{T}(l,d)=\{\begin{array}{c} l\times E_{elec}+l\times \varepsilon_{fs}\times d^{2},\;d<d_{0}\\ l\times E_{elec}+l\times \varepsilon_{mp}\times d^{4},\;d\geq d_{0} \end{array}$$

where *E_elec_* is the energy consumed by a sensor node to transmit or receive 1-bit data, and *Ɛ*_*fs*_ and *Ɛ*_*mp*_ are two amplifier coefficients of free-space model and multi-path fading model respectively. The threshold distance *d*_0_ is calculated by Equation (2):

(2) $$d_{0}=\sqrt{\varepsilon_{fs}/\varepsilon_{mp}}$$

where *Ɛ*_*fs*_ and *Ɛ*_*mp*_ are two parameters of the amplifier. When *d < d*_0_, the energy consumption of the sensor nodes uses the Free-space model, and the amplifier parameter is *Ɛ*_*fs*_. When *d* ≥ *d*_0_, the energy consumption of the sensor nodes uses the multi-path fading model, the amplifier parameter is *Ɛ*_*mp*_. 

In this paper, the maximum transmission distance of a node is controlled not to exceed *d*_0_, which ensures that nodes in the same cluster are within the transmission range of the proposed PSO-based uneven dynamic clustering method.

## 4. Overview of PSO

Before presenting the proposed algorithm, we give an outline of the particle swarm optimization (PSO) (Kennedy et al. 1995) algorithm [35]. PSO is based on a swarm of particles of a predefined number (say *N_p_*). Each particle *P_i_* (1 ≤ *I* ≤ *N_p_*) provides a complete solution to a multidimensional optimization problem. Dimension *D* of all the particles is equal. Particle *P_i_* has position *X_i_*_,*d*_ (1 ≤ *d* ≤ *D*) and velocity *V_i_*_,*d*_ in the *d*th dimension of the multidimensional space. Let the *i*th particle *P_i_* of the population be represented by Equation (3) as follows.

(3) $$P_{i}=[X_{i,1}(t),X_{i,2}(t),X_{i,3}(t),\ldots ,X_{i,D}(t)]$$

A fitness function is used to evaluate each particle to judge its quality of the solution to the problem. The personal best called *Pbest_i_* is the best position of each particle *P_i_*. The global best called *Gbest* is the best position of all particles*_._* In order to reach the global best position, each particle *P_i_* follows its own best, i.e., *Pbest_i_* and *Gbest* to update its own velocity and position. In each iteration, its velocity *V_i_*_,*d*_ and position *X_i_*_,*d*_ in dimension *D* is updated by using Equations (4), (5), respectively.

(4) $$V_{i,d}(t)=w\times V_{i,d}(t-1)+c_{1}\times r_{1}\times (Pbest_{i,d}-X_{i,d}(t-1))+c_{2}\times r_{2}\times (Gbest_{d}-X_{i,d}(t-1))$$

(5) $$X_{i,d}(t)=X_{i,d}(t-1)+V_{i,d}(t-1)$$

(6) $$w=w_{\max}-\frac{w_{\max}-w_{\min}}{T_{R}}\times t$$

where *T*_R_ is the maximum number of iterations, *w* (*w*_max_ = 0.9, *w*_min_ = 0.4) is self-adapting parameter, *c_1_* and *c_2_* (0 ≤ *c*_1_*, c*_2_ ≤ 2) are the acceleration coefficients, and r_1_ and r_2_ (0 < r_1_, r_2_ < 1) are the randomly generated values. The update process is repeated until an acceptable value of *G_best_* is obtained or a fixed number of iterations (*t_max_*) is reached. After getting a new updated position, the particle evaluates the fitness function and updates *P_besti_* as well as *G_best_* for the minimization problem as follows.

(7) $$Pbest_{i}=\{\begin{array}{ll} P_{i}, & if(Fitness(P_{i})<Fitness(Pbest_{i}))\\ Pbest_{i}, & otherwise \end{array}$$

(8) $$Gbest=\{\begin{array}{ll} P_{i}, & if(Fitness(P_{i})<Fitness(Gbest))\\ Gbest, & otherwise \end{array}$$

**Figure 1:**
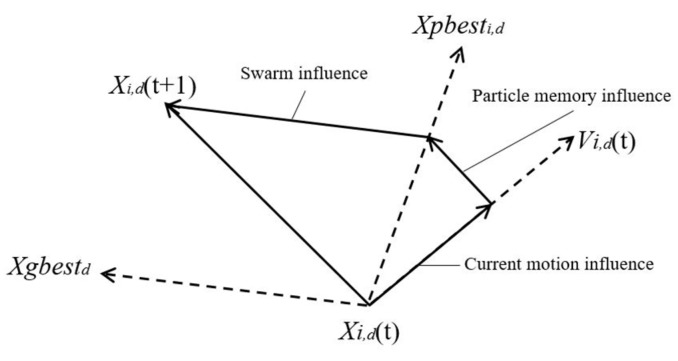
Update process of particle position in multidimensional space

Figure 1 shows how a particle explores in the multi-dimensional search space to achieve a global best solution. A particle *P_i_* occupies position *X_i,d_*(*t*) with velocity *V_i,d_*(*t*) at a point of time and it is moving in a certain direction. Later the particle changes the direction and moves to another position using its memory. It then changes its direction again by the influence of the swarm and occupies a new position *X_i,d_*(*t+*1).

**Figure 2:**
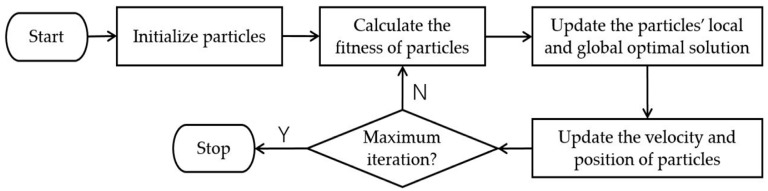
The workflow of PSO.

After a number of iterations, the particles will find the optimal solution in the searching space. The workflow of PSO is illustrated as Figure 2.

## 5. Proposed Algorithm

Similar to the existing hierarchical routing protocols, the operation of PUDCRP also is broken up into rounds. Each round is divided into a set-up phase and a steady-state phase. In the set-up phase, an improved PSO is used to determine the circular area where the candidate CH nodes are located. The PSO-based method operates at the BS. CH nodes are selected by a multi-objective fitness function. Non-CH nodes join the cluster where the nearest CH node is located. In the steady-state phase, a connecting line aided route construction method was proposed to achieve an energy efficient routing.

### 5.1. Terminologies

For the ease of understanding of the proposed algorithm, we first define some terminologies as follows.

1. *S*: The set of sensor nodes, i.e., *S*= {*s*_1_, *s*_2_, …, *s_n_*}.
2. *A*: The area of the network.
3. *d*_0_: The threshold distance.
4. *E*_0_: The initial energy of nodes.
5. *E_i_*: The residual energy of sensor node *s_i_*, 1 ≤ *I* ≤ *n*.
6. *P*: The set of particles, i.e., *P*= {*P*_1_, *P*_2_, …, *P_Np_*}, *N_p_* is the number of particles.
7. *D*: The dimension of particle characteristics.

### 5.2. Particle Representation and Initialization

In PSO, a particle swarm represents a complete solution. For the clustering process of the proposed algorithm, a particles represents optimal positions of the center of the circular areas where candidate CH nodes are located. Each component *P_i_*(*t*) = (*X_i,_*_1_(*t*), *X_i,_*_2_(*t*)) = (*x_i_*(*t*), *y_i_*(*t*)) denotes the coordinates of the center of a circular area. 

#### 5.2.1. Determination Radius of Circular Area

In multi-hop routing protocols, the CH nodes closer to the BS undertake more data forwarding tasks, which causes nodes near the BS area to die prematurely and generates undetectable hotspots. This is the so-called hotspot problem. To address the problem, an effective solution is to make the clusters closer to the BS smaller and make the clusters farther away from the BS larger. Small clusters near the BS are with fewer nodes and have short transmission distance to the BS. Therefore, this approach can compensate for the energy consumption of the nodes near the BS for forwarding data from the other CH nodes. Since in the proposed algorithm the distribution of the circular areas determines the location of CH nodes, the area of the circular areas near the BS should be smaller than the ones farther away from the BS, which can help to achieve the reasonable distribution of the above clusters to a certain extent. The radius of a circular area is calculated by Equation (9).

(9) $$R_{i}=\frac{dis_{i}}{dis_{\max}}\times (R_{\max}-d_{1})+d_{1},d_{1}\leq R_{i}\leq R_{\max}$$

where *R_i_* is the radius of the *i*th circular area, *dis_i_* is the distance between the center of the *i*th circular area and the BS, *dis*_max_ is the maximum distance between circular area centers and the BS, and *R*_max_ is the maximum radius of the circular areas. The maximum radius *R*_max_ is *d*_0_/2 in this paper. *d*_1_ is the minimum radius of circular areas, which is calculated by Equation (10).

(10) $$d_{1}=\sqrt{\frac{A}{\pi n}}$$

The value of *d*_1_ is the average radius of the area covered by a node in the network, which can ensure that the node closest to the BS can form a cluster.

#### 5.2.2. Determination Optimal Number of Circular Areas

**Figure 3:**
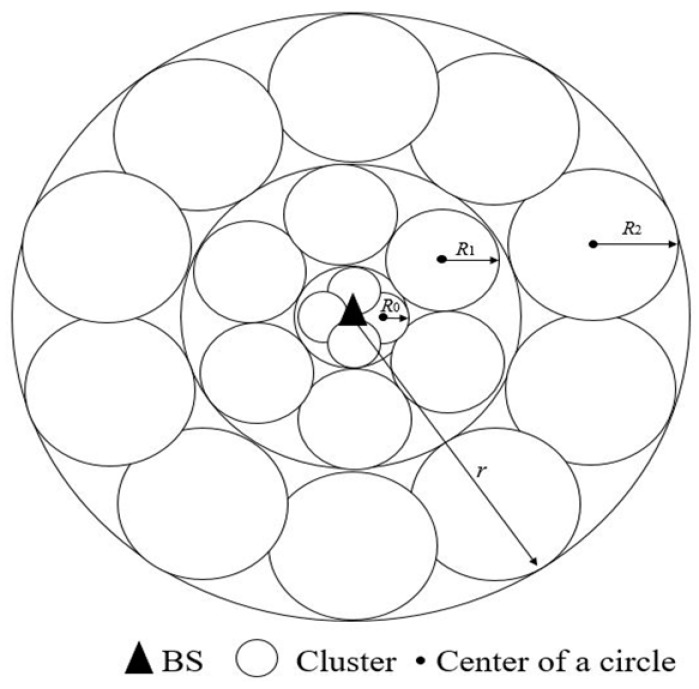
Uniform distribution by layer of circular areas.

The proper number of clusters is essential for clustering effectiveness, otherwise the network cannot benefit from clustering advantages. The optimal number of clusters is defined as C. If the value of *C* is too large, there will be many circular areas that overlap. If the value of *C* is too small, the circular area cannot cover as much of the network environment as possible. In this paper, in order to determine the C value, it is assumed that the clusters distribute the entire network environment evenly by layer, as shown in the Figure 3. There are partial overlaps between the circular areas in the figure. Because the network area cannot be filled with circles, and partial overlap of the circular areas can offset the unfilled network area. *r* is the sum of 2*R_i_* which is calculated by Equation (11). The radius of the circle of the *k*th layer is *R*_k_ (assuming that the network area is divided into *K* layers). The number of circles of the *k*th layer is *n*_k_. According to the above conditions, Equation (12) can be obtained.

(11) $$r=\sum_{i=0}^{K}2R_{i}$$

(12) $$A\approx \sum_{k=1}^{K}n_{k}\times \pi R_{k}^{2}$$

where *A* is the area of the whole network environment. 

Thus, the optimal circular area number *C* can be obtained by cyclic calculation. The following is the calculation process for calculating the *C* value. The algorithm to calculate the optimal circular area number is illustrated in Algorithm 1.
**Algorithm 1: The calculation process of optimal circular area number *C*****Input:** The radius of cluster: *R* = *d*_1_/* R is initialized to *d*_1_*/. The area of the network: *A*. The maximum distance between circular area centers and the BS: *dis*_max_. The farthest distance from the base station to the boundary of the circle where *R* has been calculated: *r*=0/* *r* is initialized to 0. *r* is the sum of 2*R_i_* which are calculated */**Output:** the optimal number of circular areas: C1. ***while*** A>0 *do*2. $A=A-\pi \left[{\left(2R+r\right)}^{2}-r^{2}\right]$3. $C=C+\frac{\pi {\left(2R+r\right)}^{2}-\pi r^{2}}{\pi R^{2}}$4. r=r+2R5. $R=\frac{r(R_{\max}-d_{1})+d_{1}\times dis_{\max}}{dis_{\max}-R_{\max}+d_{1}}$6. ***endwhile***7. C=C×H /* *H* is determined by the location of the BS*/

**Figure 4:**
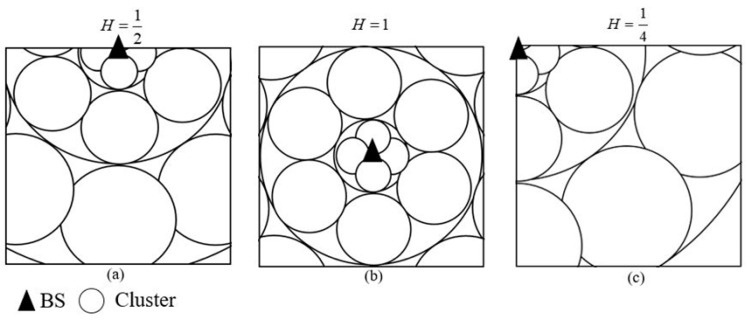
Different location of the BS in the monitoring area. (**a**) the BS is located on the boundary of the monitoring area, (**b**) the BS is located in the monitor area, (**c**) the BS is located on a vertex of the rectangle network area.

*H* is determined by the location of the BS. As shown in Figure 4, *H* in (a) where the BS is located on the boundary of the monitoring area is 1/2. *H* in (b) where the BS is located in the monitor area is 1. *H* in (c) where the BS is located on a vertex of the rectangle network area is 1/4. *H* depends on the angle between the two boundaries intersecting the BS.

### 5.3. Derivation of Fitness Function

The proposed PSO-based clustering algorithm is clustered according to node distribution. The fitness function is used to determine the distribution of the circular areas where the candidate CH nodes are located. The derivation of the fitness function depends on the two parameters: coverage rate and Intersection-over-Universal.

#### 5.3.1. Coverage Rate

Coverage rate is the ratio of the number of nodes in a circular area where candidate CH nodes are located to the total number of nodes. Obviously, the more nodes that are covered, the more candidate CH nodes can be obtained, so that local optimization can be avoided when selecting CH nodes. Moreover, it can avoid the situation where CH nodes are gathered in a certain corner. Therefore, we need to maximize the coverage rate.

(13) $$r_{Cov}=\frac{n_{Cov}}{n}$$

where *n_Cov_* is the number of nodes covered by the circular areas and *n* is the number of nodes in the network. 

#### 5.3.2. Intersection-Over-Universal

**Figure 5:**
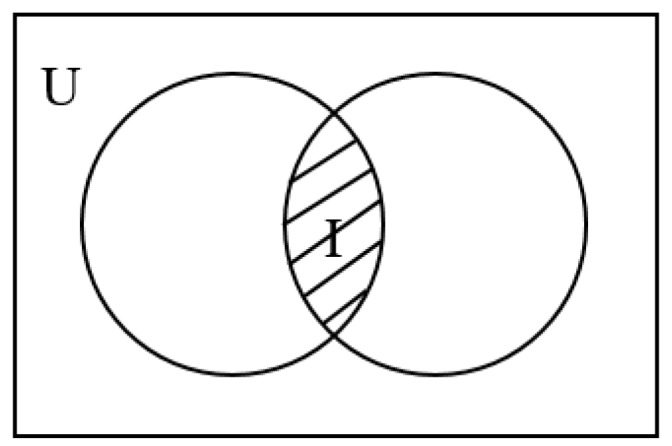
Intersection set *I* and universal set *U*.

Based on the concept of Intersection-over-Union in target detection [36], we propose an Intersection-over-Universal (*IoU*). *IoU* is the ratio of the number of candidate CH nodes in the overlapping portion of the circular area to another circular area to the total number in the network. Figure 5 shows the intersection set *I* and universal set *U*. The more nodes in the overlapping portion among the circular areas where candidate CH nodes are located, the more likely that only one shared CH node is selected among these circular areas. It causes the CH node to bear a heavy forwarding load during the transmission phase. Excessive overlap of the circular areas also reduces the coverage of the candidate CH nodes. Therefore, it is wise to minimize *IoU*. That is, we need to maximize its reciprocal.

(14) $$IoU=\frac{n_{Inter}}{n}$$

where, *n_Inter_* is the number of nodes in the overlapping portion of the circular areas.

**Figure 6:**
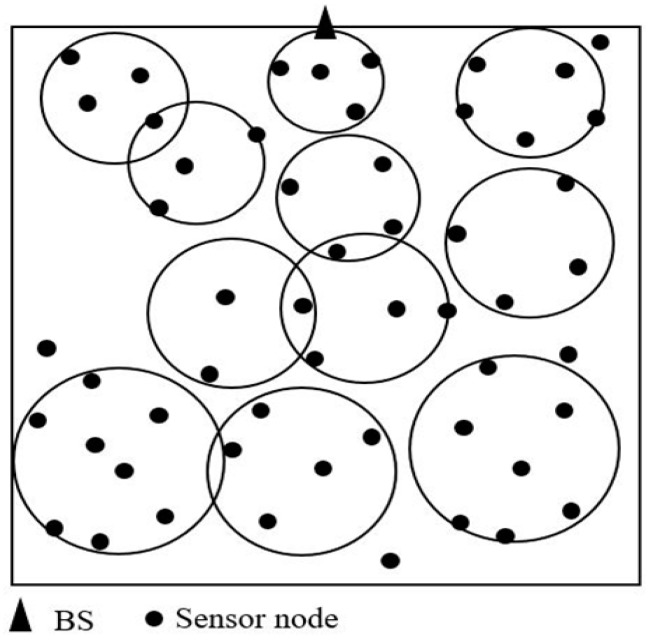
Schematic diagram of the distribution of circular areas.

After iterations, if a circular area’s *IoU* is greater than a certain threshold *T* (0 < *T* < 1, *T* = 0.7 in this paper), the center of this circular area should be deleted from the global best positions. The circular area where the center is located will also be deleted. Hence, the number of CH nodes is less than or equal to *C*. Figure 6 is the schematic diagram of the distribution of the circular areas. The circular areas cannot cover all the nodes in the network. We select CH nodes based on these circular areas. Since the circular areas are formed according to the number of nodes, the area of the whole network and the distribution of nodes, we can achieve a reasonable distribution of CH nodes. The reasonable distribution can efficiently balance the energy consumption of the whole network.

**Figure 7:**
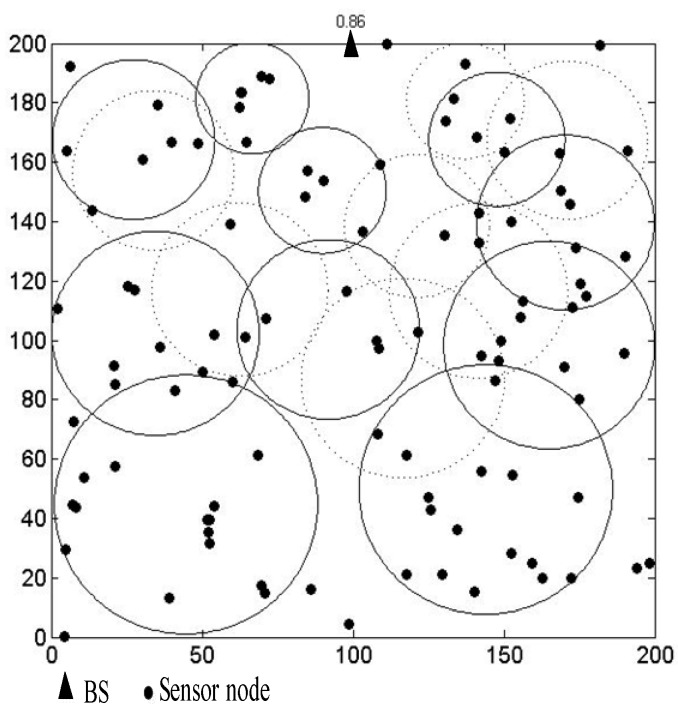
The circular area distribution of the proposed algorithm in the simulation experiment.

**Figure 8:**
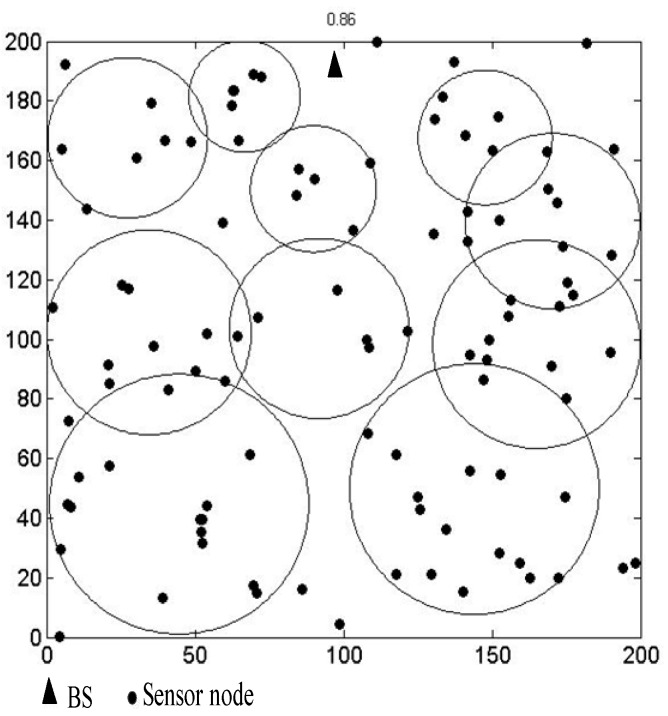
The circular area (*IoU* < 0.7) distribution of the proposed algorithm in the simulation experiment.

Figure 7 shows the circular area distribution of the proposed algorithm in simulation experiments. The network environment is a 200 m × 200 m area with 100 nodes. The dotted circle in Figure 7 is the circular area that needs to be deleted because their *IoU* is greater than 0.7. The solid circles are the circular areas obtained after iterations. The number 0.86 at the top of the figure is the final node coverage after iterations. Figure 8 shows the circular areas with *IoU* less than 0.7 in the network after iterations. As can be seen from Figure 7 and Figure 8, the circular areas cover as many nodes as possible. The distribution of the circular areas is based on the distribution of nodes. The selection of CH nodes is based on the distribution of the circular areas, the residual energy of the nodes, the distance from the nodes to the BS, and the number of neighbor nodes covered in the communication range. The distribution of CH nodes directly selected by multi-objective functions [37] may be too dense or too sparse. In addition, the circular areas close to the BS are smaller than the ones farther away from the BS, which is beneficial to balance the energy consumption of the network.

#### 5.3.3. Proposed Fitness Function

In order to determine the optimal positions of the circular areas in our PSO method, it is best to maximize the linear combination of the above two parameters instead of maximizing them individually, since the two parameters do not conflict with each other. Therefore, we use the following fitness function Equation (15) to determine *G_best_*.

(15) $$Fitness=\alpha \times r_{Cov}+\frac{1-\alpha }{IoU},0<\alpha <1$$

where, *α* is 0.9 in this paper, which is determined by experiments. Because the algorithm removes the circular areas with high *IoU*. Hence, the coverage of the circular areas is mainly considered in the fitness function.

For a single circular area, there is no concept of *IoU*. Therefore, in our PSO approach, the fitness function to determine *P_besti_* is the absolute value of the difference between the actual number of nodes covered by the circular area and the number of ideal coverage nodes.

(16) $$fitness_{i}=\left|n_{Cov_{i}}-n_{ideal_{i}}\right|,0<i<m$$

where *n_Covi_* is the number of the nodes covered in a circular area and *n_ideali_* is the ideal number of the nodes covered in a circular area. *m* is the number of circular areas.

*n_ideali_* in a circular area is related to the density of nodes in the network and the size of the circular area. *n_ideali_* is calculated by Equation (17).

(17) $$n_{ideal_{i}}=A_{i}\times \frac{n}{A}$$

where *A_i_* is the area of the *i*th circular area and *A* is the area of the whole network.

(18) $$A_{i}=\pi R_{i}^{2}$$

where, *R_i_* is the radius of the *i*th circular area.

In each iteration, the velocity and the positions of particles are updated using Equations (4) and (5). 

### 5.4. Set-Up Phase

After determining circular areas where the candidate CH nodes are located, CH nodes are selected by a multi-objective function. The CH nodes in each circular area are determined according to the residual energy of nodes, the distance from the nodes to the BS, and the number of neighbor nodes covered in the communication range. The multi-objective function is as follows.

(19) $$Weight_{ij}=\omega_{1}\times \frac{E_{ij}}{E_{0}}+\omega_{2}\times \frac{n_{nb_{i}}}{n}+\omega_{3}\times \frac{d_{\min_{j}}}{d_{s_{i}}},0\leq i\leq n,0\leq j\leq m$$

where, we assign each factor the corresponding coefficient *w*_1_, *w*_2_ and *w*_3_, weighing the importance of each factor to CH election. *w*_1_ + *w*_2_ + *w*_3_ = 1, 0 ≤ *w*_1_, *w*_2_, *w*_3_ ≤1, *E_ij_* is the residual energy of a candidate CH node *s_i_* in the *j*th circular area, *E_0_* is the initial energy of nodes, *n_nb__i_* is the number of neighbor nodes in the communication range of the node *s_i_*, *d_min__j_* is the minimum distance between the BS and candidate CH nodes in the *j*th circular area, and *d_si_* is the distance between the candidate CH node *s_i_* and the BS. *E_ij_* divided by *E_0_*, *n_nbi_* divided by *n*, and *d_min__j_* divided by *d_si_* are normalized to adjust their values in the range [0,1]. The purpose of normalization is to adjust the values measured on different scales into a common scale so that there will be the same impact when multiple objectives are superposed. The node with the largest *Weight_ij_* in the candidate circular area is selected as the CH node.

**Figure 9:**
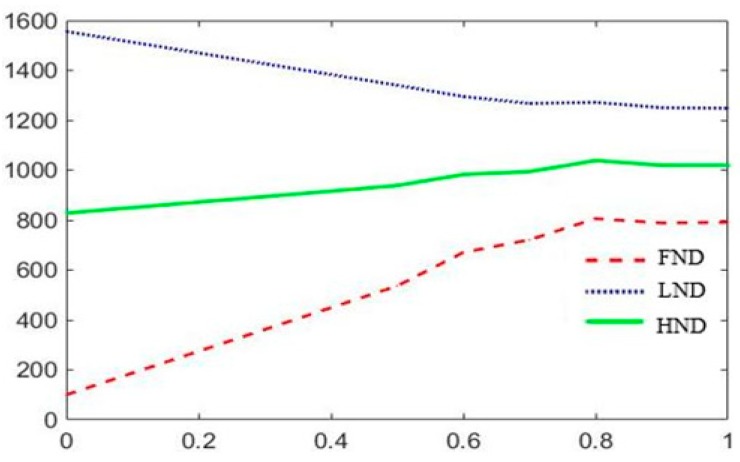
Network lifetime schematic diagram of the trend of *w*_1._

**Figure 10:**
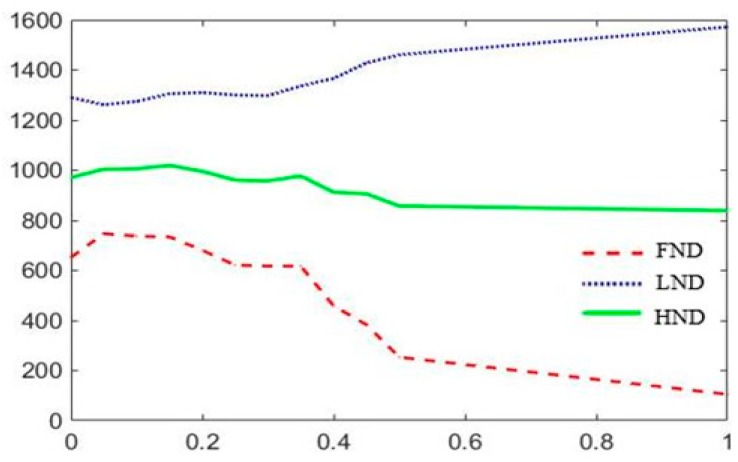
Network lifetime schematic diagram of the trend of *w*_2._

**Figure 11:**
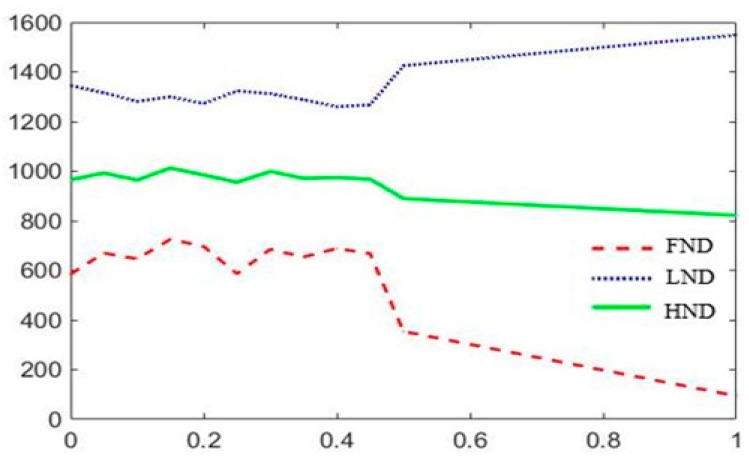
Network lifetime schematic diagram of the trend of *w*_3._

The experimental results in a 200 m × 200 m network with 100 nodes are shown in Figure 9, Figure 10 and Figure 11. Where FND indicates the round in which the first dead node occurs. HND indicates the round in which half of nodes die. LND indicates the round in which 80% nodes die. According to above experimental results, *w*_1_, *w*_2_ and *w*_3_ are set to 0.8, 0.05 and 0.15. After CH nodes are determined, each sensor node determines which cluster it wants to join by choosing the CH that requires the minimum communication energy. Once all the nodes are organized into clusters, each CH creates a schedule for the nodes in its cluster. This allows the radio components of each non-cluster head node to be turned off at any time, except at its transmission time, thereby minimizing the energy consumed by a single sensor. Once the CH node has all the data from the nodes in its cluster, it aggregates the data and then transmits the compressed data to the BS. Because the distance between the CH nodes near the BS is smaller than the ones farther away from the BS, the area of clusters which near the BS can be smaller than the ones farther away from the BS, which can alleviate the hotspot problem due to forwarding data and balance the energy consumption of the network.

The pseudocode of the PSO-based CH selection algorithm is given in Algorithm 2.


**Algorithm 2: Pseudocode of PSO-based CH selection**
**Input:** Set of sensor nodes: S = {*s*_1_, *s*_2_, …, *s*_n_}.   Optimal number of circular areas: *C*.   Number of dimensions of a particle: *D =* 2. **Output:** Optimal positions of cluster heads: CH = {*CH*_1_, *CH*_2_, …, *CH*_m_}.
Initialize particles *P_i_*, ∀_*i*_, *d*, 1 ≤ *I* ≤ *m*, 1 ≤ *d* ≤ *D**P_i_* (0) = (*X_i,_*_1_(0), *X_i,_*_2_(0)) = (*x_i_*(0), *y_i_*(0))/* The deployed positions of the sensor nodes */***for****i*=1 to *N_p_****do***Calculate fitness(*P_i_*)/*Using Equation (15) */*P_besti_* = *P_i_*
***end for***
*Gbest* = {*Pbest*_i_}, 1 ≤ *I* ≤ *N_p_****for**t* = 0 to *T_R_* **do**/*T_R_ = Max. number of iterations */***for****I* = 1 to *N_P_* ***do***Updata velocity and position of *P_i_* using Equations (4) and (5)Calculate *fitness*(*P_i_*)Calculate *Fitness*(*P_i_*)***if****fitness(P_i_)* < *fitness(Pbest_i_)* ***then****Pbest_i_* = *P_i_*
***endif***
***if****Fitness(Pi)* < *Fitness(Gbest)* ***then****Gbest* = *P_i_*
***endif***

***endfor***

***endfor***
***for****I* = 1 ***to*** *N_p_* ***do******if****IoU(P_i_)* > 0.7 ***then***delete *P_i_* from *Gbest*
***endif***

***endfor***

***while*** *Pi* ***in*** *Gbest*
Calculate *R_i_* using Equation (9)***CH**_i_* = {*S_k_*| *Weight*(*S_k_*) = max(*Weight*(*S_j_*)), ∀_*j*_,*k*,1 ≤ *j,k* ≤ *n*, *d_Sj_* ≤ *R_i_*)}/* *d_Sj_* is the distance between node *j* and *P_i_* */
***endwhile***
stop


### 5.5. Steady-State Phase

Before sending data to the BS, energy-efficient transmission routes from sensor nodes to the BS must first be established. This paper proposes a connecting line aided route construction method to address the issue. Intra-cluster communications are based on single-hop transmission. If the distance between non-CH nodes and the BS is less than *d*_0_, non-CH nodes directly send data to the BS via single-hop transmission. Each cluster member transmits data directly to its respective CH node. 

**Figure 12:**
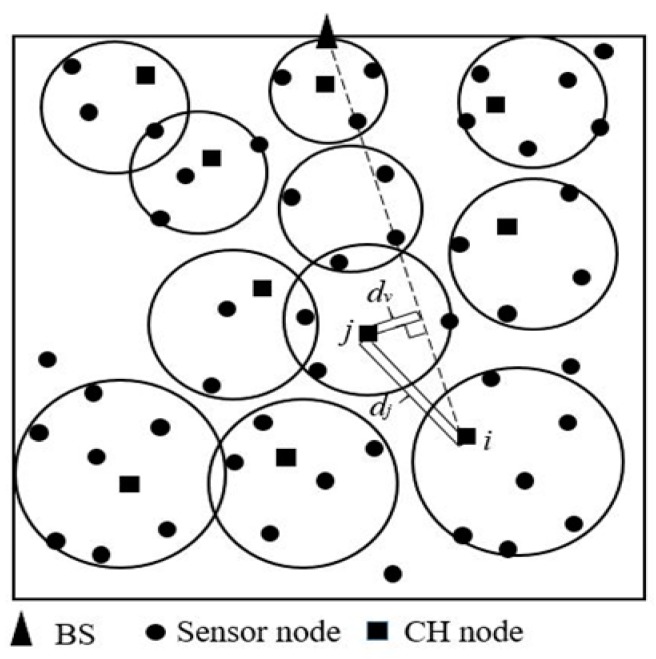
Schematic diagram of inter-cluster routing.

Based on the distance from CH nodes to the BS, inter-cluster communication uses single-hop or multi-hop transmission. If the distance between CH nodes and the BS is less than *d*_0_, CH nodes send data directly to the BS via single-hop transmission. Otherwise, CH nodes transmit data to the BS via a multi-hop route established by the connecting line aided route construction method to reduce the energy consumption of multi-hop transmission. When the CH node selects the next hop node of a multi-hop route, it comprehensively considers the distance from the next hop to itself, the connection line connecting the CH node and the BS and the residual energy of next hop. Figure 12 shows how to establish a specific transmission route from CH node *i* to the BS. 

**Figure 13:**
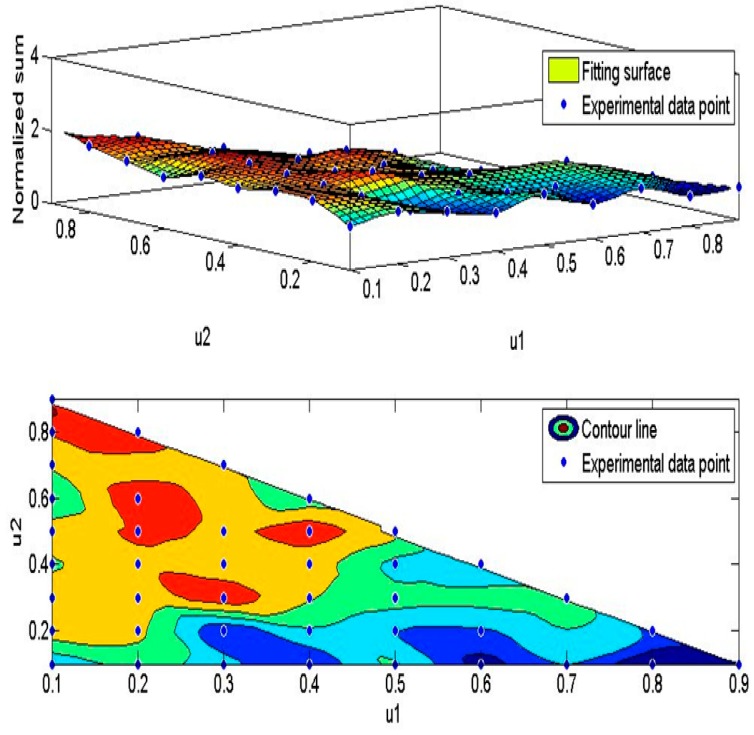
The distribution schematic of Normalization sum of FND and LND with different *u*_1_, *u*_2._

CH node *i* selects CH node *j* as the next hop. CH node *j* is selected according to Equation (20).

(20) $$W_{j}=u_{1}\times \frac{d_{v}}{d_{0}}+u_{2}\times \frac{d_{j}}{d_{0}}+u_{3}\times \frac{E_{0}}{E_{j}},\;u_{1}+u_{2}+u_{3}=1,1\leq j\leq m$$

(21) $$d_{v}=\frac{\left|(BS_{y}-CH_{yi})\times NH_{xj}+\left(CH_{xi}-BS_{x}\right)\times NH_{yj}+BS_{x}\times CH_{yi}-BS_{y}\times CH_{xi}\right|}{\sqrt{{\left(BS_{y}-CH_{yi}\right)}^{2}+{\left(CH_{xi}-BS_{x}\right)}^{2}}}$$

where *W_j_* is the weight used to determine the next hop. The node with the smallest weight is selected as the next hop. *m* is the number of CH nodes, the coordinate of CH node *i* is (*CH_xi_*, *CH_yi_*), the coordinate of the candidate next hop node *j* is (*NH_xj_*, *NH_yj_*), *d_j_* is the distance between CH*_i_* and the candidate node *j*, *d_v_* is the vertical distance from the candidate node *j* to the connecting line, which is calculated by Equation (21). *u*_1_, *u*_2_ and *u*_3_ are three corresponding coefficients. After a large number of experiments, the sums of normalized FND and normalized LND under different *u*_1_, *u*_2_ and *u*_3_ are obtained. MATLAB is used to cubic fitting to get Figure 13. According to the position of the highest contour line in Figure 13, it can be determined that *u*_1_ = 0.1 to 0.25, *u*_2_ = 0.75 to 0.8 or *u*_1_ = 0.2 to 0.25, *u*_2_ = 0.45 to 0.6, or *u*_1_ = 0.25 to 0.35, *u*_2_ = 0.3 to 0.35 or *u*_1_ = 0.35 to 0.45, *u*_2_ = 0.45 to 0.55 that FND and LND are better. In this paper, *u*_1_ = 0.2, *u*_2_ = 0.6, *u*_3_ = 1−*u*_1_−*u*_2_ = 0.2. From Figure 13, it can be seen that the distance to the next hop has a greater impact on the route selection when selecting the next hop.

**Figure 14:**
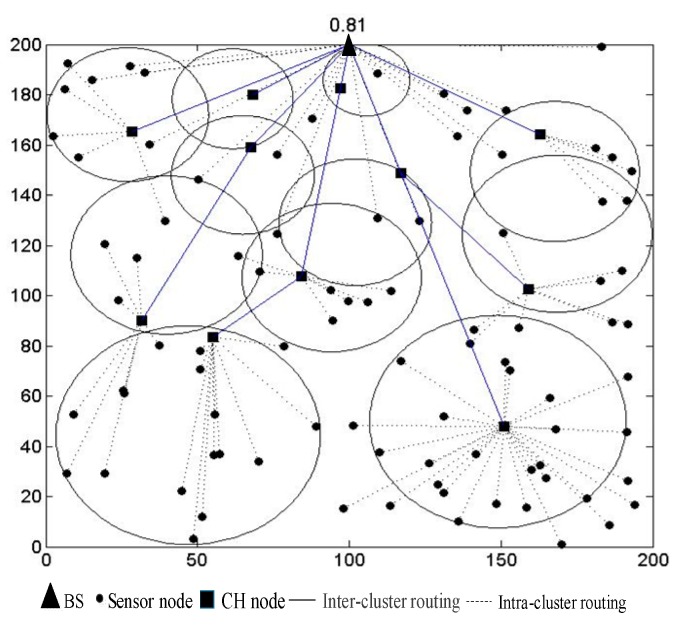
Routing diagram in simulation environment.

The candidate node with minimum weight would be selected as the next hop. Figure 14 is the routing process in simulation environment. It can be seen that the transmission path of each CH node is as close as possible to the linear distance to the BS, and the CH node close to the BS mainly acts as a relay. The routing method minimizes the energy consumption for the routes and balances the energy consumption between the CH nodes. The connecting line aided route construction method can ensure that the transmission distance between the CH nodes is as short as possible and the transmission distance from the CH nodes to the BS is as close as possible to the linear distance from the CH node to the BS. The residual energy of next hop is considered to prevent the nodes to die prematurely. Hence, the energy consumed by data transmission is significantly reduced and balanced.

## 6. Simulation and Results

To evaluate our proposed protocol, MATLAB is used to perform simulations. In order to simplify the entire simulation process, it is assumed that the network has an ideal MAC (Medium Access Control) layer. The data link communication is reliable and the energy of the BS is not restricted. In the network control process, there is no any energy load for sending control messages and receiving data. Only the energy consumption of the sensor nodes is considered during the experiments. The parameters of the network areas are pre-set.

The simulation parameters of the network area are shown in Table 1.

Simulation experiments were carried out on UCCGRA, multi-hop EEBCDA, EEMRP, CAMP, PSO-ECHS, PSO-SD and PUDCRP in the corresponding network circumstances. The proposed algorithm is based on region partitioning. Therefore, this paper mainly compares the algorithm with the algorithms based on grid region partitioning and PSO based clustering algorithm. Nodes’ death states of the protocols are shown in Table 2. 

Where FND indicates the round in which the first dead node occurs. HND indicates the round in which half of nodes die. LND indicates the round in which 80% nodes die.

Table 2 shows that the PUDCRP protocol runs more rounds than the other six protocols under the same network conditions. In the 400 m × 400 m network, compared with UCCGRA, multi-hop EEBCDA, EEMRP, CAMP, PSO-ECHS and PSO-SD, the time of the first death node in PUDCRP was delayed by 18.00%, 508.06%, 216.81%, 33.69%, 528.33% and 62.85%, respectively. HND of PUDCRP is increased by 31.95%, 61.89%, 12.67%, 61.50%, 109.42% and 52.81%, respectively. The number of running rounds of PUDCRP is increased by 48.75%, 63.21%, 68.89%, 7.36%, 74.21% and 69.81%, respectively. PUDCRP more effectively balances the energy consumption of the network and prolongs the network lifetime than the other six protocols.

**Figure 15:**
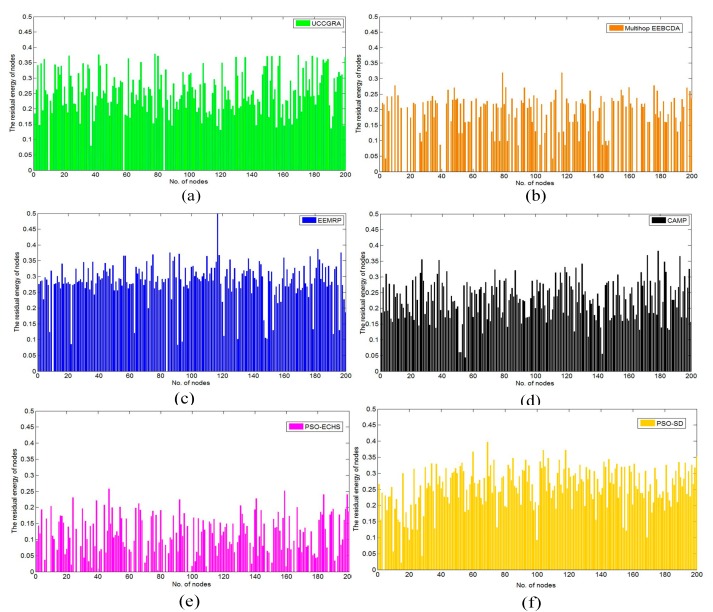

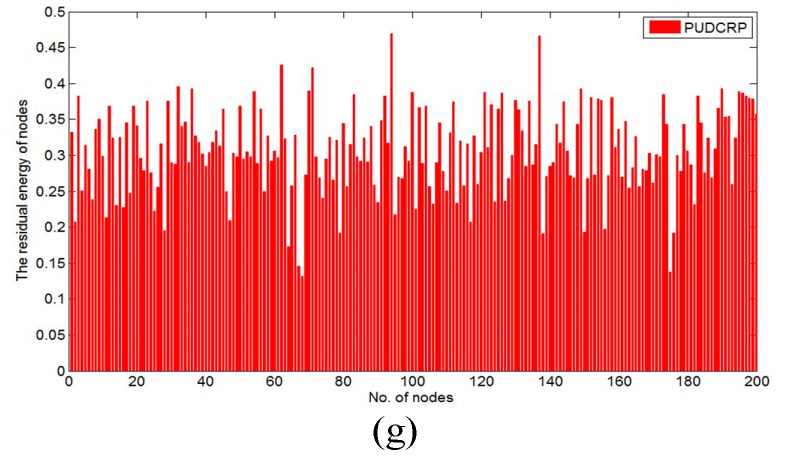
Residual energy of nodes under different algorithms in the 500th round: (**a**) UCCGRA; (**b**) Multihop EEBCDA; (**c**) EEMRP; (**d**) CAMP; (**e**) PSO-ECHS; (**f**) PSO-SD; (**g**) PUDCRP.

Table 3 shows the average and standard deviation of residual energy of nodes in the 500th round in the 400 m × 400 m network with 200 nodes. AVE indicates the average residual energy of nodes. STD indicates the standard deviation of residual energy of nodes. The more average residual energy of nodes, more effective the energy efficiency of the algorithm. Lower the standard deviation of residual energy of nodes, more balanced energy consumption. The average residual energy of nodes in PUDCRP is 22.16%, 121.30%, 8.92%, 33.73%, 197.28% and 21.05% higher than the other six algorithms, respectively. The standard deviation of residual energy of nodes in PUDCRP is 18.82%, 41.37%, 10.99%, 5.14%, 14.22% and 12.57% lower than the other six algorithms, respectively. (a)-(g) in Figure 15 are the residual energy of nodes in the 500th round in UCCGRA, multi-hop EEBCDA, EEMRP, CAMP, PSO-ECHS, PSO-SD and PUDCRP, respectively. Figure 15 visually indicates that the minimum residual energy of nodes in PUDCRP is more than 0.1 J. The minimum residual energy of nodes in other six algorithms are all less than 0.05 J. It shows that the energy consumption per node in PUDCRP is less than other algorithms. And it can be seen from Figure 15 that the energy histogram of the nodes in PUDCRP is denser than other six algorithms, which shows the balanced energy consumption of PUDCRP. The results from Table 3 and Figure 15 show that PUDCRP is much more energy-efficient and achieve more balanced energy consumption of the entire network. 

**Figure 16:**
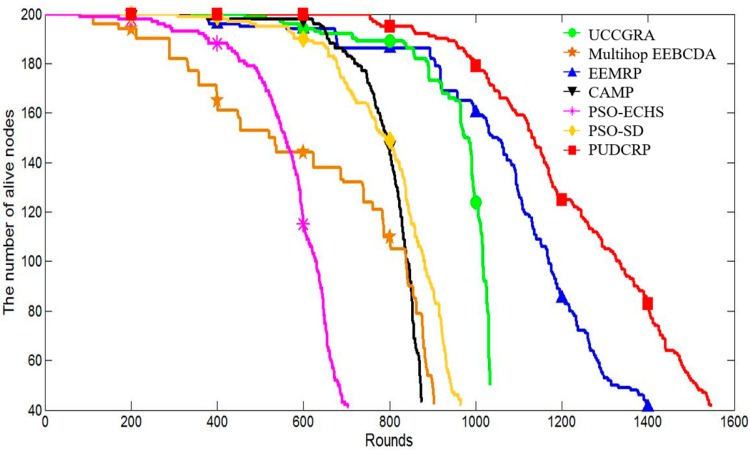
The number of surviving nodes in the 400 m × 400 m network area.

**Figure 17:**
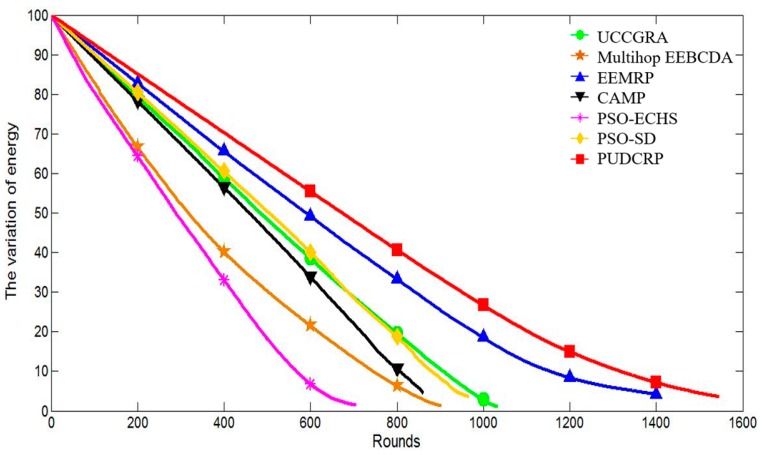
Energy consumption of the 400 m × 400 m network area.

The simulation experiments also compare the number of surviving nodes and energy consumption of the seven protocols in each round in network. The results are shown in Figure 16 and Figure 17.

Figure 16 shows how the number of surviving nodes of the seven routing protocols varies with the number of operation rounds in 400 m × 400 m networks. It can be seen that the number of surviving nodes of PUDCRP begins to decrease later than the other six protocols and the round when the first dead node occurs is significantly delayed. The number of surviving nodes in PUDCRP decreases more slowly than the other six protocols. The results mean that PUDCRP balances the energy consumption of the sensor nodes more effectively than the other protocols. 

Figure 17 shows how the energy consumption of the seven routing protocols in each round varies. The PUDCRP protocol consumes less energy than the other protocols per round. It can be concluded that compared with other six protocols, the PUDCRP can significantly reduce the energy consumption of nodes.

**Figure 18:**
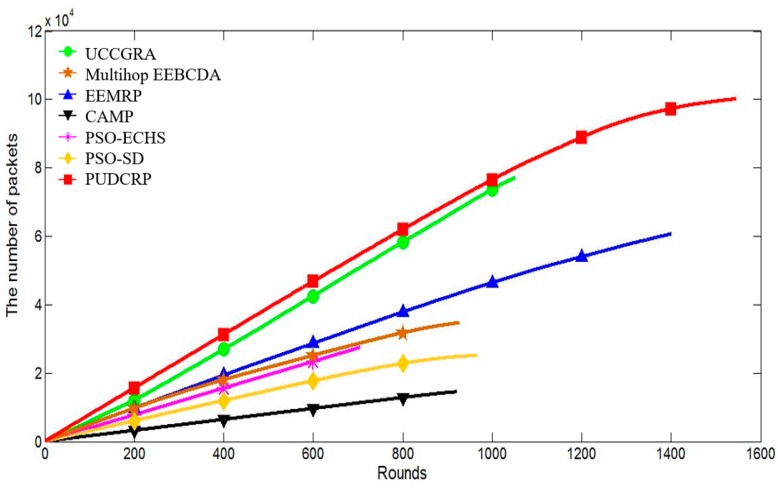
The number of packets received by the BS in the 400 m × 400 m network area.

Figure 18 shows the total number of packets received by the BS of the seven routing protocols. With increasing simulation rounds, the number of packets received by the BS is different in these protocols. 

In the PUDCRP algorithm, the BS receives far more data packets than EEMRP and other algorithms with the same rounds. In the 400 m × 400 m network, packets sent to the BS in PUDCRP are saturated at 1200th round and the BS has received 88,820 packets. However, EEMRP is saturated at 1200th round and the BS has only received 53,960 packets. The experimental results show that due to the longer network lifetime and the more balanced energy consumption, the number of packets received by the BS in PUDCRP is much higher than that of the other six protocols. The number of packets received by UCCGRA and PUDCRP during the network operation period is similar, but since the network lifetime of PUDCRP is longer than UCCGRA, PUDCRP receives more packets than UCCGRA. Balanced energy consumption delays the death of the nodes, which ensures that the number of packets received by the BS remains high for a long time. Therefore, our algorithm has a significant improvement in the data transmission performance and interactive capabilities. It also shows that under the same experimental conditions, PUDCRP can collect more data and have higher network energy efficiency.

**Figure 19:**
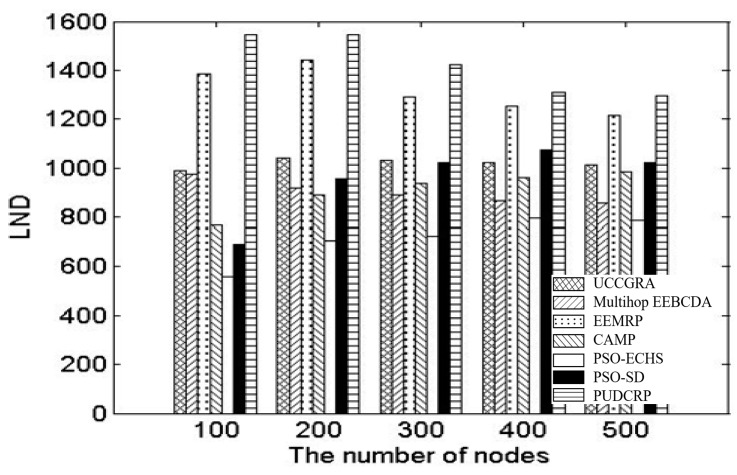
The nodes number scalability of the nodes number in 400 m × 400 m network area.

**Figure 20:**
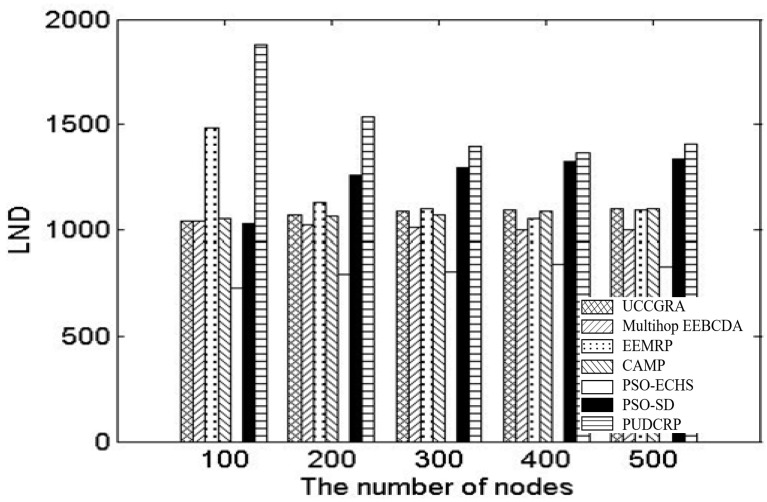
The nodes number scalability of the nodes number in 200 m × 200 m network area.

We also compared the scalability of the network nodes number and network areas of the seven protocols, the LNDs of UCCGRA, multi-hop EEBCDA, EEMRP, CAMP, PSO-ECHS, PSO-SD and PUDCRP were tested in the 200 m × 200 m and 400 m × 400 m network environments with different number of nodes, respectively. Figure 19 shows the LNDs of the seven protocols in 400 m × 400 m networks with different number of nodes. Table 4 shows specific measurement data of LNDs of the seven protocols in 400 m × 400 m networks with different number of nodes. Figure 20 shows the LNDs of the seven protocols in 200 m × 200 m networks with different number of nodes. Table 5 shows specific measurement data of LNDs of the seven protocols in 200 m × 200 m networks with different number of nodes. As can be seen from Figure 19 and Figure 20, in the network environments with different numbers of nodes, the LNDs of PUDCRP occurred significantly later than the other six protocols. The results show that PUDCRP has better scalability for network environments with different nodes and different sizes. This is due to the high energy efficiency of nodes and the balanced energy consumption of whole network achieved by the PSO-based uneven dynamic clustering method. 

Compared to multi-hop EEBCDA and EEMRP, PUDCRP has good performance in any shape (not just in a rectangular network) and size network environment. Multi-hop EEBCDA, EEMRP and other rectangular meshing clustering algorithms are more suitable for rectangular network environments. Compared with CAMP, in PUDCRP, the farther the distance between the nodes and the BS, the larger the size of the clusters, the better the hotspot problem can be alleviated. Compared with PSO-ECHS and PSO-SD, the distribution of CH nodes in PUDCRP is more reasonable, which is determined by the distribution of nodes. Among these protocols, only PUDCRP considers the distribution of nodes in the clustering process.

## 7. Conclusions

In this paper, we proposed PUDCRP, an energy-efficient multi-hop routing protocol based on particle swarm optimization (PSO) algorithm to form clusters adaptively. The PSO-based uneven dynamic clustering method divides the network area into circles with unequal sizes according to the number and distribution of nodes. The radius of the circles is determined by the distance between the center of the circles and the BS. The farther the distance from clusters to the BS, the larger the clusters, which can effectively solve the hotspot problem of WSNs. The proposed protocol improves the way clusters are created in a wireless sensor network. The key idea is to divide the network area into multiple clusters adaptively based on distribution of nodes to achieve more balanced energy consumption. Compared with the rectangular grid clustering method and the formula selection CH clustering method, the PSO-based uneven dynamic clustering method can significantly reduce the energy consumption of nodes. We further proposed a connecting line aided route construction method to improve the energy efficiency of data transmission between the BS and CH nodes. Simulation experiments showed that compared with UCCGRA, multi-hop EEBCDA, EEMRP, CAMP, PSO-ECHS and PSO-SD, PUDCRP achieves more balanced energy consumption, significantly prolongs the network lifetime, and has better scalability in both the various number of nodes and different size networks.

## Figures and Tables

**Table 1:** Simulation parameters.

Parameter	Value
Area of network	*M* × *M* (M = 200 m, 400 m)
Co-ordinate of sink	($\frac{M}{2}$, *M*)
Number of sensor nodes (*n*)	100 to 500
Initial energy of sensor nodes (*E*_0_)	0.5 J
Energy consumption on circuit (*E*_elec)_	50 nJ/bit
Free-Space channel parameter (*ε*_fs_)	10 pJ/bit/m^2^
Multi-path channel parameter (*ε*_mp_)	0.0013 pJ/bit/m^4^
Distance threshold (*d*_0_)	$\sqrt{\frac{\varepsilon_{fs}}{\varepsilon_{mp}}}$
Data packet size	4000 bits
Control packet size	50 bits
Number of iterations	2500
The particle Velocity (*v*)	−35 ≤ *v* *≤* 35
the acceleration coefficients (*c*_1_,*c*_2_)	*c*_1_ = 2, *c*_2_ = 2

**Table 2:** The average rounds of PUDCRP in the 400 m × 400 m network area (n = 200).

Death State	UCCGRA	Multi-Hop EEBCDA	EEMRP	CAMP	PSO-ECHS	PSO-SD	PUDCRP
FND	639	124	238	564	120	463	754
HND	1011	824	1184	826	637	873	1334
LND	1040	916	1441	888	733	911	1547

**Table 3:** Average and standard deviation of residual energy of nodes in the 500th round in the 400 m × 400 m network area (n = 200).

	UCCGRA	Multi-Hop EEBCDA	EEMRP	CAMP	PSO-ECHS	PSO-SD	PUDCRP
AVE	0.2509	0.1385	0.2814	0.2292	0.1031	0.2532	0.3065
STD	0.0728	0.1008	0.0664	0.0623	0.0689	0.0676	0.0591

**Table 4:** LND of different algorithms in the 400 m × 400 m network area.

Number of Nodes	100	200	300	400	500
UCCGRA	991	1040	1030	1024	1014
Multi-hop EEBCDA	974	916	888	867	856
EEMRP	1385	1441	1290	1251	1213
CAMP	767	888	935	959	985
PSO-ECHS	560	705	726	794	784
PSO-SD	691	957	1024	1074	1022
PUDCRP	1545	1547	1423	1311	1294

**Table 5:** LND of different algorithms in the 200 m × 200 m network area.

Number of Nodes	100	200	300	400	500
UCCGRA	1043	1070	1086	1096	1102
Multi-hop EEBCDA	1043	1022	1011	1002	1003
EEMRP	1482	1129	1098	1055	1092
CAMP	1053	1064	1070	1088	1099
PSO-ECHS	726	792	803	839	826
PSO-SD	1029	1257	1296	1323	1336
PUDCRP	1881	1537	1398	1364	1408

## References

[1] Djedouboum A.C., Ari A.A.A., Gueroui A., Mohamadou A., Aliouat Z. (2018). Big Data Collection in Large-Scale Wireless Sensor Networks. Sensors.

[2] Rashid B., Rehmani M.H. (2016). Applications of wireless sensor networks for urban areas: A survey. J. Netw. Comput. Appl..

[3] Amjad M., Sharif M., Afzal M.K., Kim S.W. (2016). TinyOS-new trends, comparative views, and supported sensing applications: A review. IEEE Sens. J..

[4] Rawat P., Singh K.D., Chaouchi H., Bonnin J.M. (2014). Wireless sensor networks: A survey on recent developments and potential synergies. J. Supercomput..

[5] Wei C., Yang J., Gao Y., Zhang Z. Cluster-based routing protocols in wireless sensor networks: A survey. Proceedings of the 2011 International Conference on Computer Science and Network Technology.

[6] Heinzelman W.R., Chandrakasan A.P., Balakrishnan H. Energy-efficient communication protocol for wireless microsensor networks. Proceedings of the 33rd Annual Hawaii International Conference on System Sciences.

[7] Lindsey S., Raghavendra C.S. PEGASIS: Power-efficient gathering in sensor information systems. Proceedings of the Aerospace Conference Proceedings.

[8] Younis O., Fahmy S. (2004). HEED: A hybrid, energy-efficient, distributed clustering approach for ad hoc sensor networks. IEEE Trans. Mobile Comput..

[9] Jin Y., Wang L., Kim Y., Yang X. (2008). EEMC: An energy-efficient multi-level clustering algorithm for large-scale wireless sensor networks. Comput. Netw..

[10] Jannu S., Jana P.K. (2016). A grid based clustering and routing algorithm for solving hot spot problem in wireless sensor networks. Wirel. Netw..

[11] Singh S.K., Kumar P., Singh J.P. (2018). An Energy Efficient Protocol to Mitigate Hot Spot Problem Using Unequal Clustering in WSN. Wirel. Person. Commun..

[12] Heinzelman W.B., Chandrakasan A.P., Balakrishnan H. (2002). An application specific protocol architecture for wireless sensor network. IEEE Trans. Wirel. Commun..

[13] Xia H., Zhang R.H., Yu J., Pan Z.K. (2016). Energy-efficient routing algorithm based on unequal clustering and connected graph in wireless sensor networks. Int. J. Wirel. Inf. Netw..

[14] Yuea J., Zhang W., Xiao W., Tang D., Tang J. (2012). Energy efficient and balanced cluster-based data aggregation algorithm for wireless sensor networks. Procedia Eng..

[15] Pant M., Dey B., Nandi S. A multi-hop routing protocol for wireless sensor network based on grid clustering. Proceedings of the 2015 Applications and Innovations in Mobile Computing (AIMoC).

[16] Huang J., Hong Y., Zhao Z., Yuan Y. (2017). An energy-efficient multi-hop routing protocol based on grid clustering for wireless sensor networks. Clust. Comput..

[17] Singh S., Kumar P., Singh J. (2017). A Survey on Successors of LEACH Protocol. IEEE Access.

[18] Eletreby R.M., Elsayed H.M., Khairy M.M. CogLEACH: A spectrum aware clustering protocol for cognitive radio sensor networks. Proceedings of the 9th International Conference on Cognitive Radio Oriented Wireless Networks and Communications (CROWNCOM).

[19] Latiwesh A., Qiu D. Energy efficient spectrum aware clustering for cognitive sensor networks: CogLeach-C. Proceedings of the 10th International Conference on Communications and Networking in China (ChinaCom).

[20] Arumugam G.S., Ponnuchamy T. (2015). EE-Leach: Development of energy-efficient LEACH protocol for data gathering in WSN. EURASIP J. Wireless Commun. Netw..

[21] Sajwan M., Gosain D., Sharma A.K. (2018). CAMP: Cluster aided multi-path routing protocol for wireless sensor networks. Wirel. Netw..

[22] Myoupo J.F., Nana B.P., Tchendji V.K. (2018). Fault-tolerant and energy-efficient routing protocols for a virtual three-dimensional wireless sensor network. Comput. Electr. Eng..

[23] Ari A.A.A., Labraoui N., Yenké B.O., Gueroui A. (2018). Clustering algorithm for wireless sensor networks: The honeybee swarms nest-sites selection process based approach. Int. J. Sens. Netw..

[24] Yalçın S., Erdem E. (2019). Bacteria Interactive Cost and Balanced-Compromised Approach to Clustering and Transmission Boundary-Range Cognitive Routing in Mobile Heterogeneous Wireless Sensor Networks. Sensors.

[25] Karaboga D., Okdem S., Ozturk C. (2012). Cluster based wireless sensor network routing using artificial bee colony algorithm. Wirel. Netw..

[26] Rao P.C.S., Jana P.K., Banka H. (2017). A particle swarm optimization based energy efficient cluster head selection algorithm for wireless sensor networks. Wirel. Netw..

[27] Kuila P., Jana P.K. (2014). Energy efficient clustering and routing algorithms for wireless sensor networks: Particle swarm optimization approach. Eng. Appl. Artif. Intell..

[28] Xiang W., Wang N., Zhou Y. (2016). An energy-efficient routing algorithm for software-defined wireless sensor networks. IEEE Sens. J..

[29] Wang J., Gao Y., Liu W., Sangaiah A.K., Kim H.J. (2019). An Improved Routing Schema with Special Clustering Using PSO Algorithm for Heterogeneous Wireless Sensor Network. Sensors.

[30] Kaswan A., Singh V., Jana P.K. (2018). A multi-objective and PSO based energy efficient path design for mobile sink in wireless sensor networks. Pervasive Mobile Comput..

[31] Abdul Latiff N.M., Tsimenidis C.C., Sharif B.S. Energy-aware clustering for wireless sensor networks using particle swarm optimization. Proceedings of the 2007 IEEE 18th International Symposium on Personal, Indoor and Mobile Radio Communications.

[32] Singh B., Lobiyal D.K. (2012). A novel energy-aware cluster head selection based on particle swarm optimization for wireless sensor networks. Hum.-Centric Comput. Inf. Sci..

[33] Hamida E.B., Chelius G. A line-based data dissemination protocol for wireless sensor networks with mobile sink. Proceedings of the 2008 IEEE International Conference on Communications.

[34] Shen J., Wang A., Wang C., Hung P.C.K., Lai C.F. (2017). An Efficient Centroid-Based Routing Protocol for Energy Management in WSN-Assisted IoT. IEEE Access.

[35] Eberhart R.C., Kennedy J. A new optimizer using particle swarm theory. Proceedings of the sixth international symposium on micro machine and human science. In Proceedings of the Sixth International Symposium on Micro Machine and Human Science, MHS’95.

[36] Jiang B., Luo R., Mao J., Xiao T., Jiang Y., Ferrari V., Hebert M., Sminchisescu C., Weiss Y. (2018). Acquisition of Localization Confidence for Accurate Object Detection. Computer Vision—ECCV 2018. Lecture Notes in Computer Science.

[37] Han G., Zhang L. (2018). WPO-EECRP: Energy-Efficient Clustering Routing Protocol Based on Weighting and Parameter Optimization in WSN. Wirel. Person. Commun..

